# Host–virus relationships: a sum of many battles

**DOI:** 10.1002/2211-5463.13420

**Published:** 2022-06-01

**Authors:** Marcelo López‐Lastra

**Affiliations:** ^1^ Laboratorio de Virología Molecular Departamento de Enfermedades Infecciosas e Inmunología Pediátrica Instituto Milenio de Inmunología e Inmunoterapia Escuela de Medicina Pontificia Universidad Católica de Chile Santiago Chile

## Abstract

The spread of pathogenic viruses implies host infection, replication, and virus dissemination. In each step, viruses have to overcome the host defenses designed to neutralize the threat they pose. The host–virus relationship represents a constant multistage battle for power as the host/cell does not voluntarily give in to the viral enemy. Upon infection, cells recognize viral pathogen‐associated molecular patterns, activating the innate antiviral defenses. As such, during most of the replication cycle, the virus has to deal with the cellular antiviral response. At this point, it should not be forgotten that viruses are obligate intracellular parasites and thus are entirely dependent on the host cell for their replication. This dependency has pushed viruses to evolve unorthodox strategies to subvert and repurpose cellular factors and processes required for efficient replication. Even if a virus has the potential to be successful at each step necessary for its spread, this does not mean it has won the war against the host. Another threat to viruses is represented by antiviral drugs designed to diminish their survival and promote the host's wellbeing. This editorial outlines the contents of this special ‘In the Limelight’ issue of *FEBS Open Bio* focused on Virology. The section contains four review articles, each focused on a particular aspect of virus–host interaction, including the antiviral response, subversion of the host translational machinery, repurposing of cellular factors, and the development of antiviral drugs.

**Abbreviations**
HSF1: heat shock factor 1
PAMPs: pathogen‐associated molecular patterns
RBP: RNA‐binding proteins
vRNAs: viral messenger RNAs

Viruses are obligate intracellular parasites, which depend on cells for their replication [1]. In consequence, viruses have evolved mechanisms to ensure that their replication can be achieved in an efficient and, in some instances, a cell‐type‐specific manner [1]. Taking control of a cell to guarantee efficient virus progeny production is far from easy. The innate immune response is a critical barrier that has to be surmounted by a virus to allow its propagation [1]. Upon infection, the cell recognizes viral pathogen‐associated molecular patterns (PAMPs), triggering cellular antiviral response mechanisms [2]. The first review by Deymier et al. [3] focuses on the interferon‐stimulated gene 20 kDa protein (ISG20), one example of a broad spectrum cellular antiviral factor used by the cell to counteract viral invasion. The review describes how ISG20 is induced in response to the cell's ability to recognize PAMPs and the molecular basis of its antiviral function. ISG20 has RNase activity targeting viral RNAs. In addition, ISG20 targets deaminated viral DNA and inhibits the translation of viral messenger RNAs (vRNAs). Viruses that antagonize the innate immune response can initiate and maintain viral gene expression. However, viruses lack components of the translational apparatus. Hence, the translation of the vRNA exclusively relies on the cellular translational machinery, which includes ribosomes, tRNAs, and the limited eukaryotic translation initiation factors pool [4]. To express its proteins, the vRNA battles with the host mRNA for the control of the host translational machinery. A small amount of vRNA has to initially compete with high concentrations of cellular mRNAs for the components of the translational machinery. Viruses have evolved unorthodox strategies to overcome this asymmetric competition and subvert the cellular machinery required for protein synthesis, guiding it preferentially to the vRNA [4]. Various strategies used by viruses to usurp the host's translational machinery rely on their ability to modify the cellular environment and repurpose the function of many cellular RNA‐binding proteins (RBP) [5, 6]. The second review from Francisco‐Velilla et al. [7] describes how picornaviruses overcome the asymmetrical competition with cellular mRNA to recruit the host's translational machinery using noncanonical molecular strategies. Picornaviruses have evolved an RNA structure named the internal ribosome entry site to initiate translation independently of factors strictly required by the cellular mRNA, such as the mRNAs 5′cap modification. The review also describes how picornaviruses target important initiation factors and subvert and repurpose the use of cellular RBPs, which usually do not function in cellular mRNA translation, to enhance viral protein synthesis [7]. Therefore, during infection, picornaviruses reprogram the host's translational machinery towards the preferential synthesizing viral proteins. The third review by Reyes et al. [8] describes the intricate relationship between viruses and a particular cellular transcription factor, the heat shock factor 1 (HSF1). The HSFs are induced during stress conditions such as heat stress orchestrating the heat shock response in cells [9]. The review first highlights that HSF1, the most studied member of the HSF family of proteins, plays an important role in favoring or diminishing viral replication, before describing how HSF1 exerts differential functions on different viral entities; as such, HSF1 has been proposed as a potential target for antiviral drug development [8].

A successful infection implies that a virus has managed to overcome all host antiviral responses and has been capable of taking control of the cell processes required for its efficient replication [1]. Successful infection by a pathogenic virus is associated with a virus‐induced disease. Even though the war against the virus might seem lost, efforts are constantly being made to develop antiviral drugs and vaccines to assist the host in this crude battle for power. The fourth review from Caceres et al. [10] highlights the efforts to control pathogenic viruses by developing antiviral drugs. The review focuses on influenza viruses, which are responsible for more than 3 million severe cases with 300 000–650 000 deaths every year [11]. It describes the currently FDA‐approved antiviral drugs and drugs under clinical trials, highlighting the molecular basis for the drug function. Furthermore, this review describes the relevance of the animal models used for drug validation.

As the Editor of this issue, I thank all authors and reviewers for the time devoted and their excellent contributions to this ‘In the Limelight’ section. I hope the provided insights into the world of host–virus relationships will be of interest to all of our readership.

## Conflict of interest

The authors declare no conflict of interest.

## Author contribution

MLL wrote the editorial.
